# Power Analysis and Sample Size Planning in ANCOVA Designs

**DOI:** 10.1007/s11336-019-09692-3

**Published:** 2019-12-10

**Authors:** Gwowen Shieh

**Affiliations:** Department of Management Science, National Chiao Tung University, Hsinchu, 30010 Taiwan, ROC

**Keywords:** general linear hypothesis, omnibus test, power, sample size

## Abstract

The analysis of covariance (ANCOVA) has notably proven to be an effective tool in a broad range of scientific applications. Despite the well-documented literature about its principal uses and statistical properties, the corresponding power analysis for the general linear hypothesis tests of treatment differences remains a less discussed issue. The frequently recommended procedure is a direct application of the ANOVA formula in combination with a reduced degrees of freedom and a correlation-adjusted variance. This article aims to explicate the conceptual problems and practical limitations of the common method. An exact approach is proposed for power and sample size calculations in ANCOVA with random assignment and multinormal covariates. Both theoretical examination and numerical simulation are presented to justify the advantages of the suggested technique over the current formula. The improved solution is illustrated with an example regarding the comparative effectiveness of interventions. In order to facilitate the application of the described power and sample size calculations, accompanying computer programs are also presented.

**Supplementary Information:**

The online version contains supplementary material available at 10.1007/s11336-019-09692-3.

## Introduction

The analysis of covariance (ANCOVA) was originally developed by Fisher (1932) to reduce error variance in experimental studies. Its essential nature and principal use were well explicated by Cochran (1957) and subsequent articles in the same issue of *Biometrics*. The value and use of ANCOVA have also received considerable attention in social science, for example, see Elashoff (1969), Keselman et al. (1998), and Porter and Raudenbush (1987). Comprehensive introduction and fundamental principles can be found in the excellent texts of Fleiss (2011), Huitema (2011), Keppel and Wickens (2004), Maxwell and Delaney (2004), and Rutherford (2011). It is essential to note that ANCOVA provides a useful approach for combining the advantages of two highly acclaimed procedures of analysis of variance (ANOVA) and multiple linear regression. The extensive literature shows that it is one of the major methods of statistical analysis in applied research across many scientific fields.

The importance and implications of statistical power analysis in scientific research are well demonstrated in Cohen (1988), Kraemer and Blasey (2015), Murphy et al. (2014), and Ryan (2013), among others. Accordingly, it is of great practical value to develop theoretically sound and numerically accurate power and sample size procedures for detecting treatment differences within the context of ANCOVA. There are numerous published sources that address statistical theory and applications of power analysis for ANOVA and multiple linear regression. Specifically, various algorithms and tables for power and sample size calculations in ANOVA have been presented in the classic sources of Bratcher et al. (1970), Pearson and Hartley (1951), Scheffe (1961), and Tiku (1967, 1972). The corresponding results for multiple regression and correlation, especially the distinct notion of fixed and random regression settings, were given in Gatsonis and Sampson (1989), Mendoza and Stafford (2001), Sampson (1974), and Shieh (2006, 2007). However, relatively little research has attempted to address the corresponding issues for ANCOVA.

This lack of further discussion can partly be attributed to the simple framework and conceptual modification of Cohen (1988) on the use of ANOVA method for power evaluation in ANCOVA research. It is argued that the ANCOVA of original responses is essentially the ANOVA of the regression-adjusted or statistically controlled measurements obtained from the linear regression of unadjusted responses on the covariates that is common to all treatment groups. However, some modifications are required to account for the number of covariate variables and the strength of correlation between the response and covariate variables. Accordingly, both the error degrees of freedom and variance component are reduced. Then, the power and sample size computations in ANCOVA proceed in exactly the same way as in analogous ANOVA designs. The methodology of Cohen (1988) has become common practice for power analysis in ANCOVA settings as repeatedly demonstrated in Huitema (2011), Keppel and Wickens (2004), Levin (1997), Maxwell and Delaney (2004), and Yang et al. (1996).

It is well known that the ANOVA adopts the fundamental assumptions of independence, normality, and constant variance. The corresponding hypothesis testing and theoretical considerations are valid only if these assumptions are satisfied. The consequences of violations of independence assumption in ANOVA have been reported in Kenny and Judd (1986), Pavur and Nath (1984), and Scariano and Davenport (1987), among others. An essential assumption underlying ANCOVA is the regression coefficients associating the response variable with the covariate variables are the same for each treatment group. Therefore, the regression adjustment in Cohen’s (1988, pp. 379–380) covariance framework includes the common regression coefficient estimates derived from the multiple regression between the response and covariate variables across all treatment groups. Unlike the original responses, the adjusted responses are generally correlated and thus violate the independence of observations assumption for ANOVA. Therefore, Cohen’s (1988) procedure is intrinsically inexact, even with the technical considerations of a deflated degrees of freedom and a correlation-adjusted variance. Consequently, this prevailing method only provides approximate power and sample size calculations in ANCOVA designs. It should be stressed that no research to date has acknowledged this crucial problem and the result has most likely been interpreted as an exact solution.

Toward the goal of choosing the most appropriate methodology for ANCOVA studies, the present article focuses on the Wald tests for the general linear hypothesis of treatment effects. Under the two different assumptions of a priori specified covariate values and multinormal distributed covariate variables, the exact power functions of the Wald statistic are derived. The analytic derivations for a general linear hypothesis require the involved operations of matrix algebra and sophisticated evaluations of matrix *t* variables that have not been reported elsewhere. Detailed numerical investigations were conducted to evaluate the existing formulas for power and sample size computations under a wide range of model settings, including non-normal covariate variables. According to the analytic justification and empirical assessment, the suggested approach has a decisive advantage over the conventional method. An applied example regarding the comparative effectiveness of interventions is presented to illustrate the distinct features and practical usefulness of the proposed techniques. Computer codes are also presented to implement the recommended power calculation and sample size determination in planning ANCOVA studies.

## General Linear Hypothesis

A one-way fixed-effects ANCOVA model with multiple covariates can be expressed as

1 $$\begin{aligned} Y_{ij} =\upmu _{i} +\sum \limits _{k=1}^P {X_{kij} \upbeta _{k} +\upvarepsilon _{ij} ,} \end{aligned}$$

where $Y_{ij}$ is the score of the *j*th subject in the *i*th treatment group on the response variable, $\upmu _{i}$ is the *i*th group intercept, $X_{kij}$ is the score of the *j*th subject in the *i*th treatment group on the *k*th covariate, $\upbeta _{k}$ is the slope coefficient of the *k*th covariate, and $\upvarepsilon _{ij}$ is the independent $N(0, \upsigma ^{2})$ error with $i = 1,\ldots , G\, (\ge 2), j = 1, \ldots , N_{i}$, and $k = 1, \ldots , P\, (\ge 1)$. The least-square estimator for the *i*th intercept $\upmu _{i}$ is given by

2 $$\begin{aligned} {\hat{\upmu }}_{i} ={\bar{Y}}_{i}-\sum \limits _{k=1}^P \bar{X}_{ki\cdot }{\hat{\upbeta }}_{k,} \end{aligned}$$

where ${\bar{Y}}_{i} =\mathop \sum \limits _{j=1}^{N_i}Y_{ij}/N_{i}$, ${\hat{{\varvec{\upbeta }}}} = ({\hat{\upbeta }}_{1}, \ldots , {\hat{\upbeta }}_{P})^{\mathrm {T}} = {\mathbf{S}}_{XX}^{-1} {\mathbf{S}}_{XY}$, $\mathbf{S}_{XX} =\mathop \sum \limits _{i=1}^G \mathop \sum \limits _{j=1}^{Ni} (\mathbf{X}_{ij} - \bar{\mathbf{X}}_{i})(\mathbf{X}_{ij} - \bar{\mathbf{X}}_{i})^{\mathrm{T}}$, $\mathbf{S}_{XY} =\mathop \sum \limits _{i=1}^G \mathop \sum \limits _{j=1}^{Ni} (\mathbf{X}_{ij} - \bar{\mathbf{X}}_{i})(Y_{ij} - \bar{Y}_{i})$, $\mathbf{X}_{ij} = (X_{1ij}, {\ldots }, X_{Pij})^{\mathrm{T}}$, $\bar{\mathbf{X}}_{i }=\mathop \sum \limits _{j=1}^{Ni} \mathbf{X}_{ij}/N_{i} = (\bar{X}_{1i\cdot }, {\ldots }, \bar{X}_{Pi\cdot })^{\mathrm{T}}$, and $\bar{X}_{ki\cdot }=\mathop \sum \limits _{j=1}^{Ni} X_{kij}/N_{i}$. Accordingly, the least-squares estimators ${\hat{\upmu }}_{i}$ of $\upmu _{i}$ have the following distributions:

3 $$\begin{aligned} {\hat{\upmu }}_{i} \sim N(\upmu _{i}, \upsigma ^{2}\{1/N_{i}+\bar{\mathbf{X}}_i^{\mathrm{T}} \mathbf{S}_{XX}^{-1} \bar{\mathbf{X}}_{i}\})\hbox { and }{ Cov}({\hat{\upmu }}_{i}, {\hat{\upmu }}_{{i}^\prime } )=\upsigma ^{2}\bar{\mathbf{X}}_i^{\mathrm{T}} \mathbf{S}_{XX}^{-1} \bar{\mathbf{X}}_{{i}^\prime } \end{aligned}$$

for $i \ne i^\prime$, *i* and $i^\prime = 1,\ldots , G$. Because the covariances between regression-adjusted estimators $\{{\hat{\upmu }}_{1}, {\ldots }, {\hat{\upmu }}_{G}\}$ are generally not zero, they should not be treated as independent variables. For notational simplicity, the prescribed properties are expressed in matrix form:

4 $$\begin{aligned} \hat{\varvec{\upmu }} \sim N_{G}({{\varvec{\upmu }}}, \upsigma ^{2}{} \mathbf{V}), \end{aligned}$$

where $\hat{\varvec{\upmu }} = ({\hat{\upmu }}_{1}, {\ldots }, {\hat{\upmu }}_{G})^{\mathrm{T}}$, ${{\varvec{\upmu }}} = (\upmu _{1}, {\ldots }, \upmu _{G})^{\mathrm{T}}$, $\mathbf{V} = \mathbf{D} + \bar{\mathbf{X}}^{\mathrm{T}}{} \mathbf{S}_{XX}^{-1} \bar{\mathbf{X}}$, $\mathbf{D} = \hbox {Diag}(1/N_{1}, {\ldots }, 1/N_{G})$ is the $G \times G$ diagonal matrix with diagonal elements $\{1/N_{1}, {\ldots }, 1/N_{G}\}$, and $\bar{\mathbf{X}}= (\bar{\mathbf{X}}_{1}, {\ldots }, \bar{\mathbf{X}}_{G})$.

The adjusted group means are the expected group responses evaluated at the grand covariate means:

5 $$\begin{aligned} \upmu _i^*=\upmu _{i}+\sum _{k=1}^P {\bar{X}}_{k\cdot \cdot }\upbeta _{k}\quad \hbox { for }i = 1,\ldots , G, \end{aligned}$$

where $\bar{X}_{k\cdot \cdot }=\mathop \sum \limits _{i=1}^{G}\mathop \sum \limits _{j=1}^{Ni} X_{kij}/N_{T}$, $k = 1,\ldots , P,$ and $N_{T}=\mathop \sum \limits _{i=1}^G N_{i}$. A natural and unbiased estimator of the adjusted group mean $\upmu _i^*$ is

6 $$\begin{aligned} {\hat{\upmu }} _i^*= {\hat{\upmu }}_{i}+\sum _{k=1}^P {\bar{X}}_{k\cdot \cdot } {\hat{\upbeta }}_{k}={\bar{Y}}_{i} - \sum _{k=1}^P {\hat{\upbeta }}_{k}({\bar{X}}_{ki\cdot } - {\bar{X}}_{k\cdot \cdot }). \end{aligned}$$

Then, the least-squares estimators ${\hat{\upmu }}_i^*$ of the adjusted group means $\upmu _i^*$ have the following distributions:

$$\begin{aligned} {\hat{\upmu }} _i^*\sim N(\upmu _i^*, \upsigma ^{2}\{1/N_{i} + ({\bar{\mathbf{X}}}_{i} - \mathbf{M})^{\mathrm{T}}{} \mathbf{S}_{XX}^{-1} ({\bar{\mathbf{X}}}_{i} - \mathbf{M})\}) \end{aligned}$$

and

7 $$\begin{aligned} {Cov}({\hat{\upmu }}_{i}, {\hat{\upmu }}_{{i}^\prime } )=\upsigma ^{2}({\bar{\mathbf{X}}}_{i} - \mathbf{M})^{\mathrm{T}}{} \mathbf{S}_{XX}^{-1} ({\bar{\mathbf{X}}}_{{i}^\prime } - \mathbf{M}), \end{aligned}$$

where $\mathbf{M}=\mathop \sum \limits _{i=1}^G \mathop \sum \limits _{j=1}^{Ni} \mathbf{X}_{ij}/N_{T} = (\bar{X}_{1\cdot \cdot }, {\ldots }, \bar{X}_{P\cdot \cdot })^{\mathrm{T}}$ for $i \ne i^\prime$, *i* and $i^\prime = 1,\ldots , G$. The vector of adjusted group mean estimators $\hat{\varvec{\upmu }}^* = ({\hat{\upmu }}_1^{*}, {\ldots }, {\hat{\upmu }}_G^*)^{\mathrm{T}}$ has the distribution

8 $$\begin{aligned} {\hat{\varvec{\upmu }}}^*\sim N_{G}({{\varvec{\upmu }}}^*, \upsigma ^{2}{} \mathbf{V}^*), \end{aligned}$$

where ${\varvec{\upmu }}^* = (\upmu _1^*, {\ldots }, \upmu _G^*)^{\mathrm{T}}$, $\mathbf{V}^* = \mathbf{D} + ({\bar{\mathbf{X}}} - \mathbf{M1}_G^{\mathrm{T}})^{\mathrm{T}}{} \mathbf{S}_{XX}^{-1} ({\bar{\mathbf{X}}} - \mathbf{M1}_G^{\mathrm{T}} )$, and $\mathbf{1}_{G}$ is a $G \times 1$ column vector of all 1’s.

To test the general linear hypothesis about treatment effects or adjusted mean effects in terms of

9 $$\begin{aligned} \hbox {H}_{0}{:} \,\mathbf{C}\upmu ^* = \mathbf{0}_{c}\hbox { versus H}_{1}{:}\, \mathbf{C}\upmu ^* \ne \mathbf{0}_{c}, \end{aligned}$$

where **C** is a $c \times G$ contrast matrix of full row rank and $\mathbf{0}_{c}$ is a $c \times 1$ null column vector, the Wald test statistic is of the form

10 $$\begin{aligned} W^* = (\mathbf{C}\hat{\varvec{\upmu }}^*)^{\mathrm{T}}(\mathbf{CV}^*\mathbf{C}^{\mathrm{T}})^{-1}(\mathbf{C}\hat{\varvec{\upmu }}^*)/\{(G - 1){\hat{\upsigma }}^{2}\} \end{aligned}$$

where ${\hat{\upsigma }}^{2} = {SSE}/\upnu$, ${SSE} = \mathop \sum \limits _{i=1}^G \mathop \sum \limits _{j=1}^{Ni} (Y_{ij} - \bar{Y}_{i})^{2} - \mathbf{S}_{XY}^{\mathrm{T}} \mathbf{S}_{XX}^{-1} \mathbf{S}_{XY}$, and $\upnu =N_{T} - G - P$. Note that the contrast matrix is confined to satisfy $\mathbf{C1}_{G} = \mathbf{0}_{c}$. Hence, the general linear hypothesis of $\hbox {H}_{0}{:}\, \mathbf{C}\upmu ^* = \mathbf{0}_{c}$ versus $\hbox {H}_{1}{:} \,\mathbf{C}\upmu ^* \ne \mathbf{0}_{c}$ is equivalent to

11 $$\begin{aligned} \hbox {H}_{0}{:}\, \mathbf{C}{\varvec{\upmu }} = \mathbf{0}_{c}\hbox { versus H}_{1}{:}\, \mathbf{C}{\varvec{\upmu }} \ne \mathbf{0}_{c}. \end{aligned}$$

Also, the Wald test statistic can be rewritten as

12 $$\begin{aligned} W^* = (\mathbf{C}\hat{\varvec{\upmu }})^{\mathrm{T}}(\mathbf{CVC}^{\mathrm{T}})^{-1}(\mathbf{C}\hat{\varvec{\upmu }})/\{(G - 1)\hat{\upsigma }^{2}\}. \end{aligned}$$

The Wald-type test has great practical and pedagogical appeal than the test procedure under the full-reduced-model formulation. Because of its simplicity and generality, the associated properties are derived and presented in the subsequent illustration. Under the null hypothesis with $\mathbf{C}{\varvec{\upmu }} = \mathbf{0}_{c}$, the test statistic $W^*$ has an *F* distribution

13 $$\begin{aligned} W^* \sim F(c, \upnu ), \end{aligned}$$

where $F(c, \upnu )$ is an *F* distribution with *c* and $\upnu$ degrees of freedom, $\upnu =N_{T} - G - P$, and $N_{T}=\mathop \sum \limits _{i=1}^G N_{i}$. Hence, $H_{0}$ is rejected at the significance level $\upalpha$ if $W^* > F_{c,\,\upnu ,\,\upalpha }$, where $F_{c,\,\upnu ,\,\upalpha }$ is the upper $(100\cdot \upalpha )$th percentile of the *F* distribution $F(c, \upnu )$. For fixed covariate values of $\{\mathbf{X}_{ij}, j = 1, {\ldots }, N_{i}$ and $i = 1,\ldots , G\}$, the test statistic $W^*$ has the general distribution

14 $$\begin{aligned} W^* \sim F(c, \upnu , \Lambda ), \end{aligned}$$

where $F(c, \upnu , \Lambda )$ is a non-central *F* distribution with *c* and $\upnu$ degrees of freedom and non-centrality parameter

15 $$\begin{aligned} \Lambda = (\mathbf{C}{\varvec{\upmu }})^{\mathrm{T}}(\mathbf{CVC}^{\mathrm{T}})^{-1}(\mathbf{C}{\varvec{\upmu }})/\upsigma ^{2}. \end{aligned}$$

The associated power function of the general linear hypothesis is readily obtained as

16 $$\begin{aligned} \Psi (\Lambda )=P\{F(c, \upnu , \Lambda ) > F_{c,\,\upnu ,\,\upalpha }\}. \end{aligned}$$

## Random Covariate Models

The prescribed statistical inferences about the general linear hypothesis are based on the conditional distribution of the covariate outcomes. As noted in Gatsonis and Sampson (1989), Mendoza and Stafford (2001), and Sampson (1974), the actual values of covariates cannot be known in advance just as the primary responses. It is vital to treat the covariates as random variables and to derive the distribution of the test statistic over possible values of the covariate variables. Moreover, Elashoff (1969) and Harwell (2003) emphasized that the statistical assumptions underlying the ANCOVA include the random assignment of subjects to treatments and the covariate variables are independent of the treatment effects. Moreover, the normal covariate setting is commonly employed to provide a fundamental framework for analytical derivation and theoretical discussion in ANCOVA studies as in Elashoff (1969) and Harwell (2003). Thus, it is constructive to assume the covariates have independent and identical normal distribution

17 $$\begin{aligned} \mathbf{X}_{ij} \sim N_{P}({{\varvec{\uptheta }}}, {{\varvec{\Sigma }}}), \end{aligned}$$

where ${{\varvec{\uptheta }}}$ is a $P \times 1$ vector and ${{\varvec{\Sigma }}}$ is a $P \times P$ positive-definite variance–covariance matrix for $i = 1,\ldots , G$, and $j = 1, {\ldots }, N_{i}$.

Under the multinormal distribution of $\{\mathbf{X}_{ij} \sim N_{P}({{\varvec{\uptheta }}}, {{\varvec{\Sigma }}}), j = 1, {\ldots }, N_{i}$ and $i = 1,\ldots , G\}$, it is straightforward to show (Gupta and Nagar 1999, Theorem 2.3.10 and Theorem 3.3.6) that $\mathbf{Z} = \bar{\mathbf{X}}{} \mathbf{C}^{\mathrm{T}}(\mathbf{CDC}^{\mathrm{T}})^{-1/2}$ has a matrix normal distribution and $\mathbf{S}_{XX}$ has a Wishart distribution

18 $$\begin{aligned} \mathbf{Z} \sim N_{P, c}(\mathbf{0}, {\varvec{\Sigma }}\otimes \mathbf{I}_{c})\hbox { and }{} \mathbf{S}_{XX} \sim W_{P}(N_{T} - G, {\varvec{\Sigma }}), \end{aligned}$$

where $\mathbf{I}_{c}$ is an identity matrix of dimension *c*. Accordingly, both $\mathbf{T} = \{\mathbf{S}_{XX} + \mathbf{ZZ}^{\mathrm{T}}\}^{-1/2}{} \mathbf{Z}$ and $\mathbf{T}^* = \mathbf{T}{\varvec{\upxi }}$ have an inverted matrix variate *t*-distribution (Gupta and Nagar 1999, Section 4.4):

19 $$\begin{aligned} \mathbf{T} \sim {IT}_{P,C}(\upnu + 1, \mathbf{0}, \mathbf{I}_{P}, \mathbf{I}_{c})\hbox { and }{} \mathbf{T}^* \sim {IT}_{P,1}(\upnu + 1, \mathbf{0}_{P}, \mathbf{I}_{P}, \Gamma ), \end{aligned}$$

where ${\varvec{\upxi }} = (\mathbf{CDC}^{\mathrm{T}})^{-1/2}(\mathbf{C}{\varvec{\upmu }})/\upsigma$ and $\Gamma = {\varvec{\upxi }}^{\mathrm{T}}{\varvec{\upxi }} = (\mathbf{C}{\varvec{\upmu }})^{\mathrm{T}}(\mathbf{CDC}^{\mathrm{T}})^{-1}(\mathbf{C}{\varvec{\upmu }})/\upsigma ^{2}$. Moreover, $A^* = \mathbf{T}^{*^{\mathrm{T}}}{} \mathbf{T}^*/\Gamma$ has a matrix variate beta type I distribution (Gupta and Nagar 1999, Theorem 5.2.4) or a Beta distribution

20 $$\begin{aligned} A^* \sim B_1^I (P/2, (\upnu + 1)/2) \equiv \hbox {Beta}\{P/2, (\upnu + 1)/2\}. \end{aligned}$$

Following these results, standard matrix algebra shows that non-centrality parameter $\Lambda$ defined in Equation 15 has the alternative form

21 $$\begin{aligned} \Lambda = {\varvec{\upxi }}^{\mathrm{T}}(\mathbf{I}_{c} - \mathbf{T}^{\mathrm{T}}{} \mathbf{T}){\varvec{\upxi }} = \Gamma B^*, \end{aligned}$$

where $B^* = (1 - A^*) \sim \hbox {Beta}\{(\upnu + 1)/2, P/2\}$. In connection with the effect size measures in ANOVA, the first component ${\varvec{\Gamma }}$ in $\Lambda$ is rewritten as

22 $$\begin{aligned} \Gamma = N_{T}\upgamma ^{2} \end{aligned}$$

where $\upgamma ^{2}=\upsigma _{\upgamma }^{2} /\upsigma ^{2}$, $\upsigma _{\upgamma }^{2} = (\mathbf{C}{\varvec{\upmu }})^{\mathrm{T}}(\mathbf{CQC}^{\mathrm{T}})^{-1}(\mathbf{C}{\varvec{\upmu }})$, $\mathbf{Q} = \hbox {Diag}(1/q_{1}, {\ldots }, 1/q_{G})$, $q_{i}=N_{i}/N_{T}$ for $i = 1,\ldots , G$. Consequently, the non-centrality term $\Lambda$ has a useful formulation

23 $$\begin{aligned} \Lambda = N_{T}\upgamma ^{2}B^*. \end{aligned}$$

It should be pointed out that Gupta and Nagar (1999) only provides the generic definition and analytic properties of an inverted matrix variate *t*-distribution. Their results are applied and extended here to the context of ANCOVA. Accordingly, under the random covariate modeling framework, the $W^*$ statistic has the two-stage distribution

24 $$\begin{aligned} W^*{\vert }B^* \sim F(c, \upnu , \Lambda )\hbox { and }B^* \sim {Beta}\{(\upnu + 1)/2, P/2\}. \end{aligned}$$

The exact power function can be formulated as

25 $$\begin{aligned} \Psi _{E}(\Lambda ) = E_{B}[P\{F(c, \upnu , \Lambda ) > F_{c,\,\upnu ,\, \Lambda } \}], \end{aligned}$$

where the expectation $E_{B}$ is taken with respect to the distribution of $B^*$.

Notably, the omnibus test of the equality of treatment effects is a special case of the general linear hypothesis by specifying the contrast matrix as $\mathbf{C}_{D}{\varvec{\upmu }} = \mathbf{0}_{(G - 1)}$ where

26 $$\begin{aligned} \mathbf{C}_{D} = (\mathbf{1}_{(G - 1)}, -\mathbf{I}_{(G - 1)}) \end{aligned}$$

is a $(G - 1) \times G$ contrast matrix of full row rank. The component $\upgamma ^{2}$ in the non-centrality term $\Lambda$ is simplified as

27 $$\begin{aligned} \updelta ^{2}=\upsigma _{\updelta }^{2} /\upsigma ^{2}, \end{aligned}$$

where $\upsigma _{\updelta }^{2} =\sum _{i=1}^G q_{i}(\upmu _{i} - \tilde{\upmu })^{2}$ and $\tilde{\upmu }=\sum _{i=1}^G q_{i}\upmu _{i}$. The corresponding non-central component $\Lambda$ is expressed as

28 $$\begin{aligned} \Lambda _{D}=N_{T}\updelta ^{2}B^*. \end{aligned}$$

The power function of the omnibus *F* test of treatment differences is simplified as

29 $$\begin{aligned} \Psi _{E}(\Lambda _{D})=E_{B}[P\{F(G - 1, \upnu , \Lambda _{D}) > F_{(G-1),\,\upnu ,\,\upalpha }\}]. \end{aligned}$$

Note that $\upsigma _{\updelta }^{2}$ reduces to the form $\upsigma _{\updelta }^{2} =\mathop \sum \limits _{i=1}^G (\upmu _{i} - \bar{\upmu })^{2}/G$ with $\bar{\upmu }=\mathop \sum \limits _{i=1}^G \upmu _{i}/G$ when $q_{i} = 1/G$ for all $i = 1, {\ldots }, G$. Hence, $\updelta ^{2}$ has the same form as the signal to noise ratio $f^{2}$ in ANOVA (Fleishman 1980) for balanced designs. Although the prescribed application of general linear hypothesis is discussed only from the perspective of a one-way ANCOVA design, the number of groups *G* may also represent the total number of combined factor levels of a multi-factor ANCOVA design. Hence, using a contrast matrix associated with a specific designated hypothesis, the same concept and process of assessing treatment effects can be readily extended to two-way and higher-order ANCOVA designs.

## Sample Size Determination

It is essential to note that the power function $\Psi _{E}$ depends on the group intercepts $\{ \upmu _{1},\ldots , \upmu _{G}\}$ and variance component $\upsigma ^{2}$ through the non-centrality $\Lambda$ or the effect size $\upgamma ^{2}$, but not the covariate coefficients $\{ \upbeta _{1}, \ldots , \upbeta _{P}\}$ . Also, under the prescribed stochastic assumptions for the covariate variables, the multivariate normal distribution leads to the unique conditional property on a beta distribution in the general distribution of the test statistic $W^*$. Due to the fundamental property of the contrast matrix, the resulting distribution and power function do not depend on the mean vector ${\varvec{\uptheta }}$ and variance–covariance matrix ${\varvec{\Sigma }}$ of the multinormal covariate distribution. To determine sample sizes in planning research designs, the power functions $\Psi _{E}$ can be applied to calculate the sample sizes $\{ N_{{E1}},\ldots , N_{EG}\}$ needed to attain the specified power $1 - \upbeta$ for the chosen significance level $\upalpha$, contrast matrix **C**, intercept parameters $\{ \upmu _{1},\ldots , \upmu _{G}\}$, variance component $\upsigma ^{2}$, and the number of covariates *P*.

For an ANCOVA design with a priori designated sample size ratios $\{r_{1},\ldots , r_{G}\}$ with $r_{i} = N_{i}/N_{1}$ for $i = 1, \ldots , G$. The required computation is simplified to deciding the minimum sample sizes $N_{{E}{1}}$ (with $N_{Ei} = N_{{E}{1}}\cdot r_{i}, i = 2,\ldots , G)$ required to achieve the selected power level with the power functions $\Psi _{E}$. Using the embedded functions in popular software systems, optimal sample sizes can be readily computed through an iterative process. The SAS/IML (SAS Institute 2017) and R (R Development Core Team 2017) programs employed to perform the suggested power and sample size calculations are available as supplementary material. The proposed power and sample size procedures for the general linear hypothesis tests of ANCOVA subsume the results in Shieh (2017) for a single contrast test as a special case. Notably, the derivations and manipulations of an inverted matrix variate *t* are more involved than that of a Hotelling’s $T^{2}$ distribution as demonstrated in Shieh (2017).

Alternatively, a simple procedure for the comparison of treatment effects has been described in Cohen (1988, pp. 379–380). Unlike the proposed two-stage distribution, it is suggested that $W^*$ has a simplified *F* distribution

30 $$\begin{aligned} W^*\sim F(G - 1, \upnu , \Lambda _{A}), \end{aligned}$$

where $\Lambda _{A} = N_{T}\updelta ^{2}$. The corresponding power function is of the form

31 $$\begin{aligned} \Psi _{A}(\Lambda _{A}) = P\{ F(G - 1, \upnu , \Lambda _{A}) > F_{(G-1),\,\upnu ,\,\upalpha }\} . \end{aligned}$$

It is easily seen from the model assumption given in Equation 1 that $\upsigma _Y^2 = \hbox {Var}(Y_{ij}) ={\varvec{\upbeta }}^{\mathrm {T}} {\varvec{\Sigma }} {\varvec{\upbeta }} + \upsigma ^{2}$ and $\uprho = {Corr}(Y_{ij}, \mathop \sum \limits _{k=1}^{P}X_{kij}\upbeta _{k}) ={\varvec{\upbeta }} ^{\mathrm {T}} {\varvec{\Sigma }} {\varvec{\upbeta }} /\{ \upsigma _Y^2 \cdot {\varvec{\upbeta }}^{\mathrm {T}} {\varvec{\Sigma }} {\varvec{\upbeta }} \}^{{1/2}}$ where ${\varvec{\upbeta }} = (\upbeta _{1}, \ldots , \upbeta _{P})^{\mathrm {T}}$. Hence, the advantage of ANCOVA over ANOVA in the reduction of error variance from $\upsigma _Y^2$ to $\upsigma ^{2} = (1 - \uprho ^{2})\upsigma _Y^2$ by a factor $(1 - \uprho ^{2})$. For ease of illustration, the power function of the omnibus *F* test of treatment differences in ANOVA is also presented here:

32 $$\begin{aligned} \Psi _{O}(\Lambda _{O}) = P\{ F(G - 1, N_{T} - G, \Lambda _{O}) > F_{(G-1),\,(N_T-G),\,\upalpha }\} , \end{aligned}$$

where $\Lambda _{O} = (1 - \uprho ^{2})\Lambda _{A}$. With the reduction of error variance from $\upsigma _Y^2$ to $\upsigma ^{2} = (1 - \uprho ^{2})\upsigma _Y^2$, it is evident that $\Lambda _{O} \le \Lambda _{A}$. Hence, the computed power $\Psi _{O}$ is generally less than $\Psi _{A}$ when all other factors are fixed despite the marginal difference between the two error degrees of freedom $N_{T} - G$ and $\upnu = N_{T} - G - P$.

The prevailing procedure of Cohen (1988) provides a direct application of the ANOVA formula in combination with a reduced degrees of freedom and a correlation-adjusted variance. It is computationally simple because the simple formulation $\Psi _{A}$ depends only on a non-central *F* distribution. On the other hand, one critical disadvantage of this method is that the *F* distribution and the associated sample size formula do not fully take into account the distributional features of covariates. A direct comparison of the two non-centrality components in Equations 28 and 30 reveals that $\Lambda _{D} <\Lambda _{A}$ because $0< B^* < 1$. This indicates that the power function $\Psi _{A}$ tends to over-estimate the true power $\Psi _{E}$ and it also leads to an under-estimated sample size in attaining a desired power level. Notably, the suggested exact procedure is of pedagogical importance and involves a beta mixture of non-central *F* distributions. These theoretical examinations assure that the proposed technique has analytical superiority over the current method of Cohen (1988). Their practical accuracy will be demonstrated in the succeeding empirical assessments.

## Numerical Assessments

To further demonstrate the contrasting features and practical consequences of the proposed approach and existing methods, detailed empirical appraisals are conducted to examine their performance in power and sample size calculations. For ease of comparison, the numerical illustration considered in Maxwell and Delaney (2004, pp. 441-443) for sample size planning and power analysis is utilized as the fundamental framework.

In particular, Maxwell and Delaney (2004) described an ANOVA design with $G = 3$, group intercepts $\{ \upmu _{1}, \upmu _{2}, \upmu _{3}\}=\{ 400, 450, 500\}$ , and error variance $\upsigma _Y^2 = 10,000$. Then, an ANCOVA model is introduced with the inclusion of an influential covariate variable *X* with $\uprho = {Corr}(X, Y) = 0.5$ to partially account for the variance in the response variable *Y*. The corresponding unexplained error variance $\upsigma ^{2}$ in ANCOVA is reduced as $\upsigma ^{2}= (1 - \uprho ^{2})\upsigma _Y^2 = 7,500$. To detect the treatment differences, they showed that the total sample sizes required to have a nominal power of 0.80 are 63 and 48 for the balanced ANOVA and ANCOVA designs, respectively. Thus, the ANCOVA design has the potential benefits to attain the same power with nearly 25% fewer subjects than an ANOVA. It should be noted that the power formulas $\Psi _{A}$ and $\Psi _{O}$ given in Equations 31 and 32, respectively, were applied for sample size calculations in Maxwell and Delaney (2004). To show a profound implication of the sample size procedures, extensive simulation study was performed under a wide range of model configurations.

First, the number of covariates and the population correlation between the response and covariate variables are extended to $P = 1, \ldots , 10$ and $\uprho = 0.1$, 0.5, and 0.9. In each combined case of *P* and $\uprho$, the required total sample sizes $N_{TO}, N_{TA}$, and $N_{TE}$ are computed with the power functions $\Psi _{O}, \Psi _{A}$ and $\Psi _{E}$ for the ANOVA, approximate ANCOVA, and exact ANCOVA methods, respectively. Throughout this numerical investigation, the significance level and nominal power are chosen as $\upalpha = 0.05$, and $1 - \upbeta = 0.80$, respectively. Note that the effect sizes associated with $\uprho = 0.1$, 0.5, and 0.9 are $\updelta ^{2}= 0.1684$, 0.2222, and 0.8772, respectively. Second, to assess the potential impact of different and smaller effect sizes, the intercept parameters are modified as $\{ \upmu _{1}, \upmu _{2}, \upmu _{3}\}=\{ 410, 450, 490\}$ in the second set of numerical investigations. The resulting effect sizes are $\updelta ^{2} = 0.1077$, 0.1422, and 0.5614 for $\uprho = 0.1$, 0.5, and 0.9, respectively. Overall, these considerations result in a total of 60 different combined configurations. For $\{ \upmu _{1}, \upmu _{2}, \upmu _{3}\}=\{ 400, 450, 500\}$ , the computed total sample sizes $N_{T}$ are summarized in Tables 1, 2 and 3 for $\uprho = 0.1$, 0.5, and 0.9, respectively. On the other hand, the corresponding results of $\{ \upmu _{1}, \upmu _{2}, \upmu _{3}\}=\{ 410, 450, 490\}$ are presented in Tables 4, 5 and 6.

**Table 1:** Computed sample size, estimated power, and simulated power for the ANOVA, approximate ANCOVA, and exact ANCOVA methods when $G = 3$, $\upsigma ^2_Y = 10000$, $\uprho = 0.1$, $\upsigma ^2 = 9900$, $\{\upmu _1, \upmu _2, \upmu _3\} = \{400, 450, 500\}$, $\updelta ^2 = 0.1684$, Type I error $\upalpha = 0.05$, and nominal power $1 - \upbeta = 0.80$

*P*	ANOVA				Approximate ANCOVA				Exact ANCOVA			
	$N_{TO}$	Estimated power	Simulated power	Error	$N_{TA}$	Estimated power	Simulated power	Error	$N_{TE}$	Estimated power	Simulated power	Error
1	63	0.8148	0.8169	$-$ 0.0021	63	0.8185	0.8158	0.0027	63	0.8115	0.8123	$-$ 0.0008
2	63	0.8148	0.8062	0.0086	63	0.8181	0.7993	0.0188	63	0.8038	0.7995	0.0043
3	63	0.8148	0.8014	0.0134	63	0.8178	0.7922	0.0256	66	0.8176	0.8216	$-$ 0.0040
4	63	0.8148	0.7913	0.0235	63	0.8174	0.7874	0.0300	66	0.8104	0.8094	0.0010
5	63	0.8148	0.7795	0.0353	63	0.8170	0.7796	0.0374	66	0.8029	0.8026	0.0003
6	63	0.8148	0.7703	0.0445	63	0.8166	0.7773	0.0393	69	0.8166	0.8185	$-$ 0.0019
7	63	0.8148	0.7671	0.0477	63	0.8161	0.7622	0.0539	69	0.8093	0.8080	0.0013
8	63	0.8148	0.7516	0.0632	63	0.8157	0.7522	0.0635	69	0.8018	0.8038	$-$ 0.0020
9	63	0.8148	0.7412	0.0736	63	0.8152	0.7476	0.0676	72	0.8156	0.8210	$-$ 0.0054
10	63	0.8148	0.7355	0.0793	63	0.8147	0.7302	0.0845	72	0.8083	0.8049	0.0034

**Table 2:** Computed sample size, estimated power, and simulated power for the ANOVA, approximate ANCOVA, and exact ANCOVA methods when $G = 3$, $\upsigma ^2_Y = 10000$, $\uprho = 0.5$, $\upsigma ^2 = 7500$, $\{\upmu _1, \upmu _2, \upmu _3\} = \{400, 450, 500\}$, $\updelta ^2 = 0.2222$, Type I error $\upalpha = 0.05$, and nominal power $1 - \upbeta = 0.80$

*P*	ANOVA				Approximate ANCOVA				Exact ANCOVA			
	$N_{TO}$	Estimated power	Simulated power	Error	$N_{TA}$	Estimated power	Simulated power	Error	$N_{TE}$	Estimated power	Simulated power	Error
1	63	0.8148	0.9073	$-$ 0.0925	48	0.8136	0.7994	0.0142	48	0.8042	0.8006	0.0036
2	63	0.8148	0.9022	$-$ 0.0874	48	0.8130	0.7993	0.0137	51	0.8217	0.8215	0.0002
3	63	0.8148	0.9006	$-$ 0.0858	48	0.8123	0.7883	0.0240	51	0.8122	0.8062	0.0060
4	63	0.8148	0.8945	$-$ 0.0797	48	0.8115	0.7705	0.0410	51	0.8022	0.8053	$-$ 0.0031
5	63	0.8148	0.8926	$-$ 0.0778	48	0.8108	0.7592	0.0516	54	0.8201	0.8271	$-$ 0.0070
6	63	0.8148	0.8799	$-$ 0.0651	48	0.8099	0.7528	0.0571	54	0.8104	0.8177	$-$ 0.0073
7	63	0.8148	0.8737	$-$ 0.0589	48	0.8091	0.7366	0.0725	54	0.8004	0.7976	0.0028
8	63	0.8148	0.8653	$-$ 0.0505	48	0.8082	0.7196	0.0886	57	0.8185	0.8291	$-$ 0.0106
9	63	0.8148	0.8539	$-$ 0.0391	48	0.8072	0.7087	0.0985	57	0.8088	0.8157	$-$ 0.0069
10	63	0.8148	0.8526	$-$ 0.0378	48	0.8062	0.6957	0.1105	60	0.8263	0.8259	0.0004

**Table 3:** Computed sample size, estimated power, and simulated power for the ANOVA, approximate ANCOVA, and exact ANCOVA methods when $G = 3$, $\upsigma ^2_Y = 10000$, $\uprho = 0.9$, $\upsigma ^2 = 1900$, $\{\upmu _1, \upmu _2, \upmu _3\} = \{400, 450, 500\}$, $\updelta ^2 = 0.8772$, Type I error $\upalpha = 0.05$, and nominal power $1 - \upbeta = 0.80$

*P*	ANOVA				Approximate ANCOVA				Exact ANCOVA			
	$N_{TO}$	Estimated power	Simulated power	Error	$N_{TA}$	Estimated power	Simulated power	Error	$N_{TE}$	Estimated power	Simulated power	Error
1	63	0.8148	1.0000	$-$ 0.1852	15	0.8108	0.7696	0.0412	18	0.8751	0.8812	$-$ 0.0061
2	63	0.8148	1.0000	$-$ 0.1852	18	0.8934	0.8509	0.0425	18	0.8409	0.8485	$-$ 0.0076
3	63	0.8148	1.0000	$-$ 0.1852	18	0.8867	0.8026	0.0841	18	0.8007	0.8086	$-$ 0.0079
4	63	0.8148	1.0000	$-$ 0.1852	18	0.8784	0.7702	0.1082	21	0.8614	0.8628	$-$ 0.0014
5	63	0.8148	1.0000	$-$ 0.1852	18	0.8681	0.7050	0.1631	21	0.8272	0.8368	$-$ 0.0096
6	63	0.8148	1.0000	$-$ 0.1852	18	0.8548	0.6520	0.2028	24	0.8807	0.8873	$-$ 0.0066
7	63	0.8148	1.0000	$-$ 0.1852	18	0.8373	0.5948	0.2425	24	0.8516	0.8578	$-$ 0.0062
8	63	0.8148	1.0000	$-$ 0.1852	18	0.8136	0.5257	0.2879	24	0.8173	0.8263	$-$ 0.0090
9	63	0.8148	1.0000	$-$ 0.1852	21	0.9048	0.6459	0.2589	27	0.8735	0.8786	$-$ 0.0051
10	63	0.8148	1.0000	$-$ 0.1852	21	0.8904	0.5855	0.3049	27	0.8442	0.8505	$-$ 0.0063

**Table 4:** Computed sample size, estimated power, and simulated power for the ANOVA, approximate ANCOVA, and exact ANCOVA methods when $G = 3$, $\upsigma ^2_Y = 10000$, $\uprho = 0.1$, $\upsigma ^2 = 9900$, $\{\upmu _1, \upmu _2, \upmu _3\} = \{410, 450, 490\}$, $\updelta ^2 = 0.1077$, Type I error $\upalpha = 0.05$, and nominal power $1 - \upbeta = 0.80$

*P*	ANOVA				Approximate ANCOVA				Exact ANCOVA			
	$N_{TO}$	Estimated power	Simulated power	Error	$N_{TA}$	Estimated power	Simulated power	Error	$N_{TE}$	Estimated power	Simulated power	Error
1	96	0.8119	0.8143	$-$ 0.0024	93	0.8023	0.7987	0.0036	96	0.8114	0.8156	$-$ 0.0042
2	96	0.8119	0.8099	0.0020	93	0.8021	0.7925	0.0096	96	0.8063	0.8141	$-$ 0.0078
3	96	0.8119	0.8016	0.0103	93	0.8019	0.7916	0.0103	96	0.8017	0.8075	$-$ 0.0058
4	96	0.8119	0.7996	0.0123	93	0.8018	0.7863	0.0155	99	0.8109	0.8118	$-$ 0.0009
5	96	0.8119	0.7929	0.0190	93	0.8016	0.7763	0.0253	99	0.8062	0.8090	$-$ 0.0028
6	96	0.8119	0.7857	0.0262	93	0.8014	0.7802	0.0212	99	0.8014	0.7936	0.0078
7	96	0.8119	0.7836	0.0283	93	0.8012	0.7638	0.0374	102	0.8104	0.8122	$-$ 0.0018
8	96	0.8119	0.7688	0.0431	93	0.8010	0.7641	0.0369	102	0.8057	0.8094	$-$ 0.0037
9	96	0.8119	0.7729	0.0390	93	0.8008	0.7570	0.0438	102	0.8009	0.8004	0.0005
10	96	0.8119	0.7677	0.0442	96	0.8144	0.7638	0.0506	105	0.8099	0.8135	$-$ 0.0036

**Table 5:** Computed sample size, estimated power, and simulated power for the ANOVA, approximate ANCOVA, and exact ANCOVA methods when $G = 3$, $\upsigma ^2_Y = 10000$, $\uprho = 0.5$, $\upsigma ^2 = 7500$, $\{\upmu _1, \upmu _2, \upmu _3\} = \{410, 450, 490\}$, $\updelta ^2 = 0.1422$, Type I error $\upalpha = 0.05$, and nominal power $1 - \upbeta = 0.80$

*P*	ANOVA				Approximate ANCOVA				Exact ANCOVA			
	$N_{TO}$	Estimated power	Simulated power	Error	$N_{TA}$	Estimated power	Simulated power	Error	$N_{TE}$	Estimated power	Simulated power	Error
1	96	0.8119	0.9125	$-$ 0.1006	72	0.8069	0.8032	0.0037	72	0.8007	0.7979	0.0028
2	96	0.8119	0.9072	$-$ 0.0953	72	0.8066	0.7901	0.0165	75	0.8122	0.8151	$-$ 0.0029
3	96	0.8119	0.9057	$-$ 0.0938	72	0.8063	0.7850	0.0213	75	0.8062	0.8072	$-$ 0.0010
4	96	0.8119	0.9057	$-$ 0.0938	72	0.8060	0.7835	0.0225	78	0.8177	0.8215	$-$ 0.0038
5	96	0.8119	0.8916	$-$ 0.0797	72	0.8057	0.7736	0.0321	78	0.8117	0.8011	0.0106
6	96	0.8119	0.8948	$-$ 0.0829	72	0.8054	0.7681	0.0373	78	0.8054	0.8039	0.0015
7	96	0.8119	0.8922	$-$ 0.0803	72	0.8051	0.7577	0.0474	81	0.8170	0.8215	$-$ 0.0045
8	96	0.8119	0.8818	$-$ 0.0699	72	0.8048	0.7561	0.0487	81	0.8109	0.8185	$-$ 0.0076
9	96	0.8119	0.8829	$-$ 0.0710	72	0.8044	0.7306	0.0738	81	0.8047	0.8041	0.0006
10	96	0.8119	0.8770	$-$ 0.0651	75	0.8218	0.7570	0.0628	84	0.8163	0.8204	$-$ 0.0041

**Table 6:** Computed sample size, estimated power, and simulated power for the ANOVA, approximate ANCOVA, and exact ANCOVA methods when $G = 3$, $\upsigma ^2_Y = 10000$, $\uprho = 0.9$, $\upsigma ^2 = 1900$, $\{\upmu _1, \upmu _2, \upmu _3\} = \{410, 450, 490\}$, $\updelta ^2 = 0.5614$, Type I error $\upalpha = 0.05$, and nominal power $1 - \upbeta = 0.80$

*P*	ANOVA				Approximate ANCOVA				Exact ANCOVA			
	$N_{TO}$	Estimated power	Simulated power	Error	$N_{TA}$	Estimated power	Simulated power	Error	$N_{TE}$	Estimated power	Simulated power	Error
1	96	0.8119	1.0000	$-$ 0.1881	21	0.8084	0.7851	0.0233	24	0.8516	0.8516	0.0000
2	96	0.8119	1.0000	$-$ 0.1881	21	0.8035	0.7568	0.0467	24	0.8281	0.8296	$-$ 0.0015
3	96	0.8119	1.0000	$-$ 0.1881	24	0.8637	0.8023	0.0614	24	0.8022	0.8034	$-$ 0.0012
4	96	0.8119	1.0000	$-$ 0.1881	24	0.8600	0.7777	0.0823	27	0.8437	0.8500	$-$ 0.0063
5	96	0.8119	1.0000	$-$ 0.1881	24	0.8557	0.7548	0.1009	27	0.8203	0.8162	0.0041
6	96	0.8119	1.0000	$-$ 0.1881	24	0.8508	0.7160	0.1348	30	0.8586	0.8577	0.0009
7	96	0.8119	1.0000	$-$ 0.1881	24	0.8450	0.6800	0.1650	30	0.8375	0.8354	0.0021
8	96	0.8119	1.0000	$-$ 0.1881	24	0.8382	0.6346	0.2036	30	0.8141	0.8208	$-$ 0.0067
9	96	0.8119	1.0000	$-$ 0.1881	24	0.8301	0.6029	0.2272	33	0.8537	0.8593	$-$ 0.0056
10	96	0.8119	1.0000	$-$ 0.1881	24	0.8203	0.5493	0.2710	33	0.8325	0.8390	$-$ 0.0065

The sample size calculations presented in Tables 1, 2, 3, 4, 5 and 6 reveal that, as expected, the computed sample sizes of the ANOVA procedure remain identical for different number of covariates *P* when all other factors are fixed. In contrast, the sample size of the exact approach increases with increase in the number of covariates *P* and with decrease in the effect size $\updelta ^{2}$ when all other configurations are held constant. Likewise, the total sample size produced by the approximate procedure also increases with decrease in the effect size $\updelta ^{2}$. However, the reported sample sizes in Tables 1 and 2 do not vary with *P*, and the computed sample sizes marginally increase with larger *P* for the other cases in Tables 3, 4, 5 and 6. More importantly, the total sample sizes $N_{TO}, N_{TA}$, and $N_{TE}$ associated with the ANOVA, approximate ANCOVA, and exact ANCOVA procedures have a consistent order of $N_{TA} \le N_{TO} \le N_{TE}$ for all the cases in Tables 1 and 4 with $\uprho = 0.1$. The order between the two sample sizes $N_{TO}$ and $N_{TE}$ is reversed for large magnitudes of $\uprho = 0.5$ and 0.9 with $N_{TA} \le N_{TE} \le N_{TO}$ for the situations in Tables 2, 3, 5 and 6. For ease of explication, the estimated powers for the three different sample size procedures are also listed in Tables 1, 2, 3, 4, 5 and 6.

**Fig. 1:**
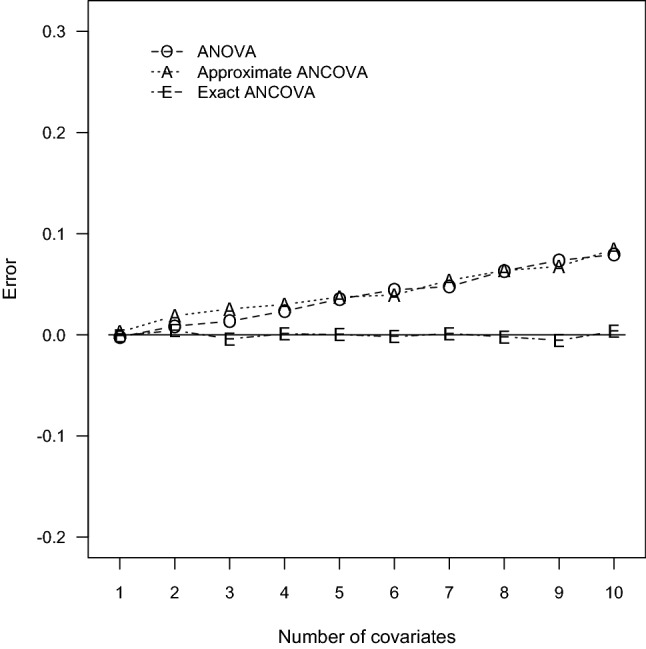
Errors of power estimation for $G = 3$ and $\uprho = 0.1$

**Fig. 2:**
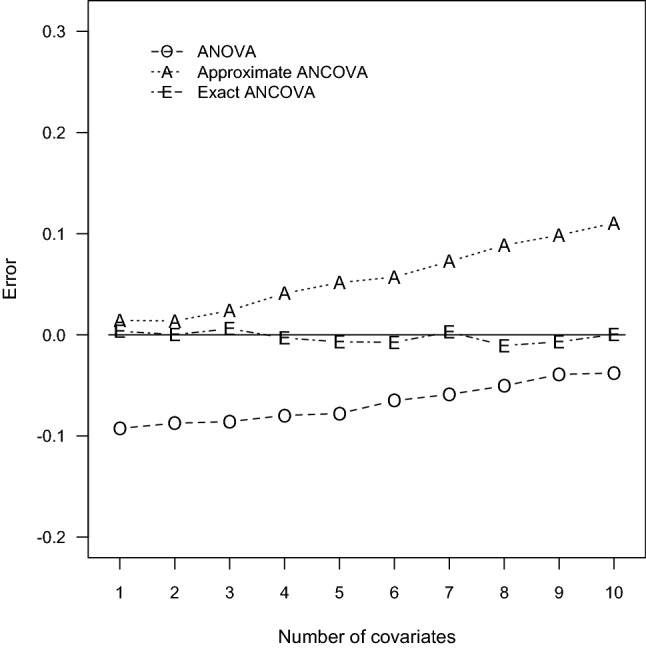
Errors of power estimation for $G = 3$ and $\uprho = 0.5$

**Fig. 3:**
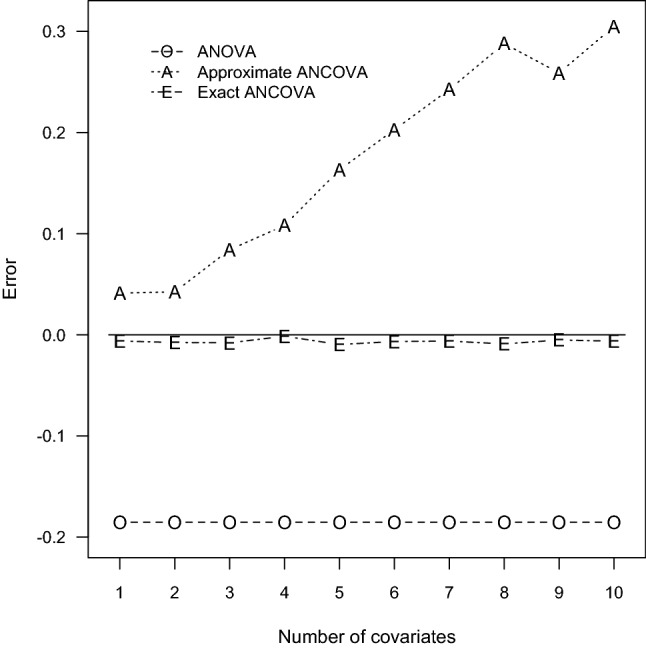
Errors of power estimation for $G = 3$ and $\uprho = 0.9$

To justify the accuracy of sample size determination, Monte Carlo simulation studies were performed for the prescribed 60 design settings. With the computed sample sizes, parameter configurations, and nominal power, estimates of the true power were computed via Monte Carlo simulation of 10,000 independent data sets. For each replicate, $N_{TO}, N_{TA}$, and $N_{TE}$ normal outcomes are generated with the ANCOVA models. Because the power function $\Psi _{E}$ is irrelevant to the mean vector $\varvec{\uptheta }$ and variance–covariance matrix $\varvec{\Sigma }$ of the designated covariate distribution, the covariates are assumed to have independent and identical multinormal distribution $N_{P}(\mathbf{0 }_{P}, \mathbf{I }_{P})$ where $\mathbf{0 }_{P}$ is a $P \times 1$ null column vector and $\mathbf{I }_{P}$ is an identity matrix of dimension *P*. The regression coefficients are chosen as $\upbeta _{1} = \ldots = \upbeta _{P} = \upbeta ^*$ and $\upbeta ^*$ is a designated value so that the resulting correlation $\uprho = 0.1$, 0.5, and 0.9. Next, the Wald test statistic $W^*$ was computed and the simulated power was the proportion of the 10,000 replicates whose test statistics $W^*$ exceed the corresponding critical value $F_{2,\,\upnu ,\,0.05}$. The simulated power and error are also summarized in Tables 1, 2, 3, 4, 5 and 6 for all the ANCOVA designs. To illustrate the contrasting behavior of the three contending techniques, the induced errors for $\uprho = 0.1$, 0.5, 0.9 in Tables 1, 2 and 3 are also plotted in Figs. 1, 2, and 3, respectively.

According to the power comparisons, the ANOVA method generally does not provide accurate sample size calculations for an ANCOVA design. Unsurprisingly, the only exceptions occurred when the number of covariates is small and the correlation between the covariates and the response variable is close to zero as in Tables 1 and 4. The approximate ANCOVA method consistently gives larger power estimate than the simulated power for all cases considered here. The discrepancy noticeably increases with the number of covariates and the magnitude of effect size. The resulting errors can be as large as 0.0845, 0.1105, and 0.3049 associated with the scenarios of $P = 10$ in Tables 1, 2 and 3, respectively. For the relative smaller effect sizes in Tables 4, 5 and 6, the performance of the approximate ANCOVA formula has improved with the errors of 0.0506, 0.0628, and 0.2710 for the cases of $P= 10$. Consequently, the overestimation problem of the power function $\Psi _{A}$ suggests that the computed sample sizes are generally inadequate to achieve the designated power level.

Regarding the accuracy of the proposed exact ANCOVA approach, the corresponding results in Tables 1, 2, 3, 4, 5 and 6 show that the differences between the estimated and simulated powers are fairly small. The largest absolute error is 0.0106 for the two cases of $P = 8$ and 5 in Tables 2 and 5, respectively. All the other 58 cases in Tables 1, 2, 3, 4, 5 and 6 have an absolute error less than 0.01. These numerical results imply that the proposed exact approach outperforms the ANOVA method and the approximate ANCOVA procedure for all design configurations considered here. Therefore, the suggested power and sample size calculations can be recommended for general use.

## An Example

A documented example of Maxwell and Delaney (2004) is presented and extended next to demonstrate the usefulness of the suggested power and sample size procedures and accompanying software programs for the omnibus test of treatment effects in ANCOVA designs.

Specifically, Maxwell and Delaney (2004, Table 9.7, p. 429) provided the data for assessing the effectiveness of different interventions for depression. There are 10 participants with random assignment in each of the three intervention groups of (1) selective serotonin reuptake inhibitor (SSRI) antidepressant medication, (2) placebo, or (3) wait list control. The measurements are the pretest and posttest Beck Depression Inventory (BDI) scores of depressive individuals. The primary interest of the ANCOVA study is on the group differences of posttest BDI measurements using the pretest BDI scores as covariates. The results show that the estimates of adjusted group means and error variance are $\{{\hat{\upmu }}_1^*,{\hat{\upmu }}_2^*,{\hat{\upmu }}_3^*\}=\{ 7.5366, 11.9849, 13.9785\}$ and ${\hat{\upsigma }}^2 = 29.0898$, respectively. The omnibus *F* test statistic of treatment differences is $W^* = 3.73$, which yields a *p*-value of 0.0376. Therefore, the test result suggests that the intervention effects are significantly different at $\upalpha = 0.05$. Although this is not the focus in the illustration of Maxwell and Delaney (2004), it can be computed from an ANOVA of posttest scores that the variance estimate is ${\hat{\upsigma }}_Y^2= 39.6185$. Hence, the sample squared correlation between the posttest and pretest BDI scores is ${\hat{\uprho }}^2 = 1 -{\hat{\upsigma }}^2{\hat{\upsigma }}_Y^2 = 1 - 29.0898/39.6185 = 0.2658$. The observed value of the ANOVA *F* test of group differences is $F^* = 3.03$ with a *p*-value of 0.0647. At the significance level 0.05, the omnibus test of no intervention group difference on the posttest BDI scores cannot be rejected. Although null hypothesis significance testing is useful in various applications, it is important to consult the recent articles of Wasserstein and Lazar (2016) and Wasserstein et al. (2019) for the recommended principles underlying proper use and interpretation of statistical significance and *p*-values.

In view of the prospective nature of advance research planning, the general guidelines suggest published findings or expert opinions can offer reliable information for the vital characteristics of future study. Accordingly, it is prudent to adopt a minimal meaningful effect size in order to enhance the generalizability of the result and the accumulation of scientific knowledge. For illustration, the prescribed summary statistics of the three-group depression intervention study are employed as population adjusted mean effects and variance component. The suggested power procedure shows that the resulting power for the omnibus test of group differences is $\Psi _{E} = 0.6145$ when the significance level $\upalpha$ equals to 0.05. Because the computed power is substantially smaller than the common levels of 0.80 or 0.90, this implies that the group sample size $N = 10$ does not provide a decent chance of detecting the potential differences between treatment groups. To determine the proper sample size, the proposed sample size computations showed that the balanced group sample sizes of 15 and 19 are required to attain the nominal power of 0.8 and 0.9, respectively. The total sample sizes $N_{T} = 45$ and 57 are substantially larger than 30 of the exemplifying design. Essentially, it requires 50% and 90% increases of the sample size to meet the common power levels of 0.80 and 0.90, respectively. These design configurations are presented in the user specifications of the SAS/IML and R programs presented in the supplemental programs. Researchers can easily identify these statements and then modify the input values in the computer code to incorporate their own model characteristics.

## Conclusions

ANCOVA provides a useful approach for combining the advantages of two widely established procedures of ANOVA and multiple linear regression. Despite the close resemblance among the three types of statistical analyses, their power computation and sample size determination are still theoretically distinct when the stochastic properties of the continuous covariates or predictors are taken into account. It is generally recognized that the use of ANCOVA may considerably reduce the number of subjects required than an ANOVA design to attain the required precision and power. For planning and evaluating randomized ANCOVA designs, an ANOVA-based sample size formula has been proposed in Cohen (1988) to accommodate the reduced error variance and degrees of freedom because of the use of effective and influential covariates. The procedure is very appealing from a computational standpoint and has been implemented in some statistical packages. However, no further analytical discussion and numerical evaluation are available to validate the appropriateness and implications of Cohen’s (1988) method in the literature.

This article aims to address the potential limitation and approximate nature of the prevailing method and to describe an alternative and exact approach for power and sample size calculations in ANCOVA designs. It is demonstrated both theoretically and empirically that the seemly exact technique of Cohen (1988) does not involve all of the covariate properties in ANCOVA. Exact power and sample size procedures are described for the general linear hypothesis tests of treatment effects under the assumption that the covariate variables have a joint multinormal distribution. The simulation results reveal that the proposed technique is superior to the current method under a wide range of ANCOVA designs. More importantly, additional numerical assessments show that the suggested power function and sample size procedure preserve reasonably good performance under various non-normal situations, such as exponential, Gamma, Laplace, Log normal, uniform, and discrete uniform distributions. Hence, the proposed two-stage distribution and power function of the Wald statistic for the general linear hypothesis tests possess desirable robust properties and are also applicable to other continuous covariate distributions in various ANCOVA designs. Consequently, the presented methodology expands the power assessment and sample size determination of Shieh (2017) for contrast analysis in ANCOVA. To enhance the practical values, computer algorithms are also provided to facilitate the recommended power calculations and sample size determinations. With respect to the importance and implementation of random sampling, the fundamental and standard sampling designs and estimation methods can be found in Thompson (2012). Heterogeneity of variance is one of the unique and problematic factors known as detrimental to the statistical inferences in ANCOVA (Harwell 2003; Rheinheimer and Penfield 2011). A potential topic for future study is to develop proper power and sample size procedures within the variance heterogeneity framework.

## Supplementary Information

Below is the link to the electronic supplementary material.

Supplementary material 1 (pdf 111 KB)
