# Erratum to: Power Analysis and Sample Size Planning in Ancova Designs

**DOI:** 10.1007/s11336-021-09780-3

**Published:** 2021-06-24

**Authors:** Gwowen Shieh

**Affiliations:** Department of Management Science, National Yang Ming Chiao Tung University, Hsinchu, 30010 Taiwan

## Erratum to: PSYCHOMETRIKA—VOL. 85, NO. 1, 101–120 MARCH 2020 10.1007/s11336-019-09692-3

The article POWER ANALYSIS AND SAMPLE SIZE PLANNING IN ANCOVA DESIGNS, written by Gwowen Shieh was originally published electronically on the publisher’s internet portal (currently SpringerLink) on 27 April 2020 without open access. With the author(s)’ decision to opt for Open Choice the copyright of the article changed on June 2021 to $\copyright$ The Author(s) 2021 and the article is forthwith distributed under the terms of the Creative Commons Attribution 4.0 International License (http://creativecommons.org/licenses/by/4.0/), which permits use, duplication, adaptation, distribution and reproduction in any medium or format, as long as you give appropriate credit to the original author(s) and the source, provide a link to the Creative Commons license and indicate if changes were made.

