# Supplementary material for: Power Analysis and Sample Size Planning in ANCOVA Designs
Source: Psychometrika. 2019 Dec 10;85(1):101–20. doi: 10.1007/s11336-019-09692-3 (PMC8225521; doi:10.1007/s11336-019-09692-3)
Supplement: Supplementary file 1 — Supplementary material 1 (pdf 111 KB) [file 11336_2019_9692_MOESM1_ESM.pdf]

Program A

SAS/IML program for computing the power for the generalized linear hypothesis tests of treatment effects

```
PROC IML;
*USER SPECIFICATION PORTION;
*DESIGNATED ALPHA;ALPHA=0.05;
*NUMBER OF GROUPS;G=3;
*NUMBER OF COVARIATES;P=1;
*GROUP SIZES;NVEC={10 10 10};
*ADJUSTED GROUP MEANS;MUVEC={7.5366 11.9849 13.9785};
*VARIANCE;SIGSQ=29.0898;
*CONTRAST COEFFICIENTS;CMAT={1 0 -1, 0 1 -1};
*END OF USER SPECIFICATION PORTION;

NUMINT=2000;DD=1E-5;
COEVEC=({1}||REPEAT({4 2},1,NUMINT/2-1)||{4 1})`;
BL=DD;BU=1-DD;
INTBL=(BU-BL)/NUMINT;BVEC=BL+(INTBL#(0:NUMINT))`;
CMU=CMAT*MUVEC`;DF1=NROW(CMAT);
NT=SUM(NVEC);QMAT=DIAG(NT/NVEC);
LGAMSQ=CMU`*INV(CMAT*QMAT*CMAT`)*CMU/SIGSQ;;
START EPOWERF;
NT=SUM(NVEC);
DF2=NT-G-P;DFX=DF2+1;B=P/DFX;FCRIT=FINV(1-ALPHA,DF1,DF2);
IF P=1 THEN DO;
TL=QUANTILE('T',DD,DFX);
TU=QUANTILE('T',1-DD,DFX);
INTL=(TU-TL)/NUMINT;TVEC=TL+(INTL#(0:NUMINT))`;
WTPDF=(INTL/3)#COEVEC#PDF('T',TVEC,DFX);
EPOWER=WTPDF`*SDF('F',FCRIT,DF1,DF2,NT#LGAMSQ/(1+B#(TVEC##2)))
;
END;ELSE DO;
BPDF=PDF('BETA',BVEC,DFX/2,P/2);
WBPDF=(INTBL/3)#COEVEC#BPDF;
EPOWER=WBPDF`*SDF('F',FCRIT,DF1,DF2,NT#LGAMSQ#BVEC);
END;
FINISH;

RUN EPOWERF;
```

```
PRINT G P LGAMSQ[FORMAT=8.4];
PRINT NVEC NT;
PRINT EPOWER[FORMAT=8.4];

QUIT;
```

Program B

SAS/IML program for computing the sample size for the generalized linear hypothesis tests of treatment effects

```
PROC IML;
*USER SPECIFICATION PORTION;
*DESIGNATED ALPHA;ALPHA=0.05;
*NOMINAL POWER;POWER=0.8;
*NUMBER OF GROUPS;G=3;
*NUMBER OF COVARIATES;P=1;
*GROUP RATIOS;RVEC={1 1 1};
*ADJUSTED GROUP MEANS;MUVEC={7.5366 11.9849 13.9785};
*VARIANCE;SIGSQ=29.0898;
*CONTRAST COEFFICIENTS;CMAT={1 0 -1, 0 1 -1};
*END OF USER SPECIFICATION PORTION;

NUMINT=2000;DD=1E-5;
COEVEC=({1}||REPEAT({4 2},1,NUMINT/2-1)||{4 1})`;
BL=DD;BU=1-DD;
INTBL=(BU-BL)/NUMINT;BVEC=BL+(INTBL#(0:NUMINT))`;
CMU=CMAT*MUVEC`;DF1=NROW(CMAT);
QMAT=DIAG(SUM(RVEC)/RVEC);
LGAMSQ=CMU`*INV(CMAT*QMAT*CMAT`)*CMU/SIGSQ;;

START EPOWERF;
NT=SUM(NVEC);
DF2=NT-G-P;DFX=DF2+1;B=P/DFX;FCRIT=FINV(1-ALPHA,DF1,DF2);
IF P=1 THEN DO;
TL=QUANTILE('T',DD,DFX);
TU=QUANTILE('T',1-DD,DFX);
INTL=(TU-TL)/NUMINT;TVEC=TL+(INTL#(0:NUMINT))`;
WTPDF=(INTL/3)#COEVEC#PDF('T',TVEC,DFX);
EPOWER=WTPDF`*SDF('F',FCRIT,DF1,DF2,NT#LGAMSQ/(1+B#(TVEC##2)))
;
END;ELSE DO;
BPDF=PDF('BETA',BVEC,DFX/2,P/2);
WBPDF=(INTBL/3)#COEVEC#BPDF;
EPOWER=WBPDF`*SDF('F',FCRIT,DF1,DF2,NT#LGAMSQ#BVEC);
END;
FINISH;
```

```
N=2;
DO UNTIL (EPOWER>POWER | N>1000);
N=N+1;NVEC=N#RVEC;
RUN EPOWERF;END;

PRINT G P LGAMSQ[FORMAT=8.4];
PRINT POWER EPOWER[FORMAT=8.4];
PRINT N NVEC NT;

QUIT;
```

Program C
R program for computing the power for the generalized linear hypothesis tests of treatment effects

```
ancova.muhtpo(0.05,3,1,c(10,10,10),c(7.5366,11.9849,13.9785),29.0898
 ,cbind(matrix(rep(1,3-1),3-1,1),-diag(3-1)))

ancova.muhtpo<-function (alpha, #DESIGNATED ALPHA
g, #NUMBER OF GROUPS
p, #NUMBER OF COVARIATES
nvec, #GROUP SAMPLE SIZES
muvec, #ADJUSTED GROUP MEANS
sigsq, #VARIANCE
cmat #CONTRAST COEFFICIENTS
) {
numint<-2000
dd<-1e-5
coevec<-c(1,rep(c(4,2),numint/2-1),4,1)
bl<-dd
bu<-1-dd
intl<-(bu-bl)/numint
bvec<-bl+intl*(0:numint)
cmu<-cmat%*%matrix(muvec,g,1)
df1<-nrow(cmat)
nt<-sum(nvec)
qmat<-diag(nt/nvec)
lgamsq<-t(cmu)%*%solve(cmat%*%qmat%*%t(cmat))%*%cmu/sigsq

epowerf<-function () {
nt<-sum(nvec)
df2<-nt-g-p
dfx<-df2+1
b<-p/dfx
fcrit<-qf(1-alpha,df1,df2)
if (p==1) {
tl<-qt(dd,dfx)
tu<-qt(1-dd,dfx)
intl<-(tu-tl)/numint
tvec<-tl+intl*(0:numint)
wtpdf<-(intl/3)*coevec*dt(tvec,dfx)
```

```
epower<-sum(wtpdf*pf(fcrit,df1,df2,nt*lgamsq/(1+b*tvec^2),lower.tail
 =FALSE))
}
else {
wbpdf<-(intl/3)*coevec*dbeta(bvec,dfx/2,p/2)
epower<-sum(wbpdf*pf(fcrit,df1,df2,nt*lgamsq*bvec,lower.tail=FALSE))
}
}

epower<-epowerf()
print("g, p, lgamsq")
print(c(g,p,lgamsq))
print("nvec, nt")
print(c(nvec,nt))
print("epower")
print(epower,digits=4)
}
```

Program D

R program for computing the sample size for the generalized linear hypothesis tests of treatment effects

```
ancova.muhtnp(0.05,0.8,3,1,c(1,1,1),c(7.5366,11.9849,13.9785),29.089
 8,cbind(matrix(rep(1,3-1),3-1,1),-diag(3-1)))

ancova.muhtnp<-function (alpha, #DESIGNATED ALPHA
power, #NOMINAL POWER
g, #NUMBER OF GROUPS
p, #NUMBER OF COVARIATES
rvec, #GROUP SAMPLE RATIOS
muvec, #ADJUSTED GROUP MEANS
sigsq, #VARIANCE
cmat #CONTRAST COEFFICIENTS
) {
numint<-2000
dd<-1e-5
coevec<-c(1,rep(c(4,2),numint/2-1),4,1)
bl<-dd
bu<-1-dd
intl<-(bu-bl)/numint
bvec<-bl+intl*(0:numint)
cmu<-cmat%*%matrix(muvec,g,1)
df1<-nrow(cmat)
qmat<-diag(sum(rvec)/rvec)
lgamsq<-t(cmu)%*%solve(cmat%*%qmat%*%t(cmat))%*%cmu/sigsq

epowerf<-function () {
nt<-sum(nvec)
df2<-nt-g-p
dfx<-df2+1
b<-p/dfx
fcrit<-qf(1-alpha,df1,df2)
if (p==1) {
tl<-qt(dd,dfx)
tu<-qt(1-dd,dfx)
intl<-(tu-tl)/numint
tvec<-tl+intl*(0:numint)
wtpdf<-(intl/3)*coevec*dt(tvec,dfx)
```

```
epower<-sum(wtpdf*pf(fcrit,df1,df2,nt*lgamsq/(1+b*tvec^2),lower.tail
 =FALSE))
}
else {
wbpdf<-(intl/3)*coevec*dbeta(bvec,dfx/2,p/2)
epower<-sum(wbpdf*pf(fcrit,df1,df2,nt*lgamsq*bvec,lower.tail=FALSE))
}
}

n<-5
epower<-0
while(epower<power & n<1000){
n<-n+1
nvec<-n*rvec
epower<-epowerf()
}
nvec<-n*rvec
nt<-sum(nvec)

print("g, p, lgamsq")
print(c(g,p,lgamsq))
print("nvec, nt")
print(c(nvec,nt))
print("epower")
print(epower,digits=4)
}
```
